# Supplementary material for: Splice-Junction-Based Mapping of Alternative Isoforms in the Human Proteome
Source: Cell Rep. Author manuscript; Available in PMC 2020 Jan 15. (PMC6961840; doi:10.1016/j.celrep.2019.11.026)
Supplement: 2 [file NIHMS1546469-supplement-2.zip › DF1/PXD006675/PulmonaryVein/PulmonaryVein_13_SVIL_RPELCTSHSETPTVDDEEK.pdf]

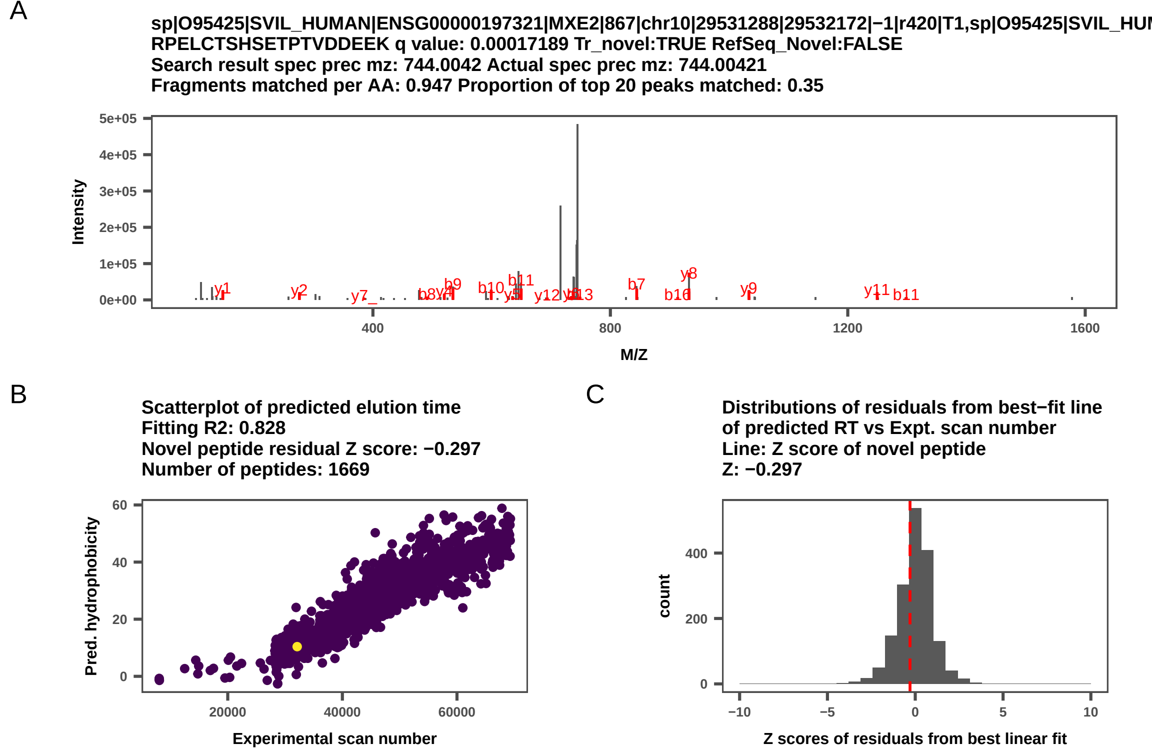
A
sp|O95425|SVIL_HUMAN|ENSG00000197321|MXE2|867|chr10|29531288|29532172|−1|r420|T1,sp|O95425|SVIL_HUM
RPELCTSHSETPTVDDEEK q value: 0.00017189 Tr_novel:TRUE RefSeq_Novel:FALSE
Search result spec prec mz: 744.0042 Actual spec prec mz: 744.00421
Fragments matched per AA: 0.947 Proportion of top 20 peaks matched: 0.35
Intensity
5e+05
4e+05
3e+05
2e+05
1e+05
0e+00
y1
y2
y7
b8
b9
b10
b11
y5
y12
b13
b7
y8
b16
y9
y11
b11
400
800
1200
1600
M/Z
B
Scatterplot of predicted elution time
Fitting R2: 0.828
Novel peptide residual Z score: −0.297
Number of peptides: 1669
Pred. hydrophobicity
60
40
20
0
20000
40000
60000
Experimental scan number
C
Distributions of residuals from best−fit line
of predicted RT vs Expt. scan number
Line: Z score of novel peptide
Z: −0.297
count
400
200
0
−10
−5
0
5
10
Z scores of residuals from best linear fit
